# Extracts

**Published:** 1883-01

**Authors:** 


### Extracts.

Painless Synovial Effusions.—A synovial effusion often takes place insidiously in the knees of women, less frequently in those of men, in consequence of what is called "a low state of health." It may be the immediate consequence of much standing or walking by persons thus conditioned; but, as a rule, it developes spontaneously, without any special cause. It may occur in both knees simultaneously, or successively, or but in one, and is usually fully established when attention is first directed to the state of the joint. It is accompanied, locally, by so slight, if any, pathological phenomena deserving the name of inflammatory, that it can scarcely be called synovitis. The familiar title "dropsy," or "water on the knee," adequately describes a state, the presence of which is a symptom, and a symptom only—as œdema of the ankles, muscæ volitantes, or a headache might be. It does not imply articular disease any more than the phenomena just mentioned imply kidney, eye, or brain disease.

It is no unusual experience to see patients with "water on the knee" maintain an unchanged condition for months, not to say years, although active treatment with iodine, blisters, or even actual cautery, has been re-enforced by absolute rest and prolonged disuse of the joint. It is alleged, in all probability, that pain is at once caused whenever, even at long intervals, attempts at exercise or use are made; and this pain, the existence of which is accepted on the bare statement of the patient, has been assumed to be the requisition for still further confinement.

It is a routine and common practice to recommend an elastic knee-cap in case of effusion. This is rarely beneficial, even if the cap be worn tight enough to serve as a pseudo splint, which I conceive to be almost its sole office. As ordinarily made it does not extend sufficiently above and below the joint; its contractile tendency shortens its already inadequate length, puckers it up, and gradually converts it into a comparatively narrow band around the articulation, which adds the discomfort of an œdema of the ankle and leg, provoked by its cord-like compression of the popliteal vessels.

A far better medium for securing the advantage of a soft and partial splint, which shall be a support to the limb, act as a check to too free use of the joint, and at the same time supply a moderate compression of the distended capsule, is found in a bandage of flannel cut "bias," that is, diagonally. The cheapest flannel should be employed. That it contains a large amount of cotton is no objection. This is not for economy's sake, but because all-wool flannel, of a good quality, is hot and clumsy, and felts and loses its elasticity when washed. The bandage should be cut at least five inches wide. It is worthless if of less than that width ; for, as it lengthens itself about one-third in application, a bandage narrower than five inches draws out into little more than a piece of tape. It must be applied with the limb straight, each turn overlapping two thirds of the previous one, and should extend from the middle of the leg to the upper third of the thigh. It is not likely to be put on too tightly, and as its elasticity permits "reversing" to be dispensed with, any patient can wind it round his limb himself. As an appliance it has the merit of being cheap and everywhere obtainable; it does not require the skill of an expert in its use; it is efficacious, and it is comfortable. * * * *

Pressure has long been resorted to and recognized as of great utility in the treatment of chronic synovial effusions, but the inadequacy of the means employed gave only imperfect results in return for its adoption. Ordinary bandages lose their hold too soon. Starched, dextrined, or silicated bandages do not follow up the shrinkage they may have started. India-rubber air or water bags are with difficulty held in place. Cotton batting beneath a bandage, though convenient and admirable in slight cases, does not afford sufficient compression. A "pure rubber" bandage, when applied tightly enough to be of service in this capacity, causes a degree of pain which renders it unendurable. Compressed sponge seems alone to supply all the desiderata.

Common, coarse, Mediteranean sponges, washed, and thoroughly dried by heat, as large or larger than the hand, and thick as they are broad, are compressed to the utmost limit which is possible in any powerful press, such as a letter-press, or between two boards screwed together. They should remain in the press twenty-four hours, and become reduced to a thickness, varying with the quality of the individual sponge, of one-half to three-quarters of an inch. The limb on which they are to be used being bandaged with a common cotton roller from the foot up, including the knee itself, so that any cutaneous irritation by the surface of the sponge may be prevented, a long and straight ham splint, lightly padded with towels, is adjusted. The compressed sponges, still in their dry state, are next applied around the knee. Three sponges usually suffice, one on each side, coming together below the joint, and a third covering the lower part of the thigh, transversely, above the patella, which they should leave uncovered. These are then firmly bound into place by a broad roller, also beginning at the ankle, and carried upward to the top of the thigh, many turns being taken over the sponges to secure them compactly in position. The bandaging completed, the sponges are then soaked with water, poured on gradually until they have absorbed so much that the pressure caused by their expansion begins to be uncomfortable. Two quarts of water are not unfrequently taken up. As this evaporates or oozes out more water is from time to time added, and fresh bandages are put on over those already in place if the latter become loose or slack. Occasionally there is pain enough provoked to call for an opiate at night, and the toes may swell, but the splint, which not only secures immobility but acts as a *point d'appui* for the bandage, takes off the direct compression from the popliteal vessels to such a degree that any anxiety in regard to arrest of circulation may be set aside.—*R. M. Hodges, M.D., in Boston Med. and Surg. Journal.*

---

Idiocy and its Treatment.—A large proportion of idiots come of stock run down and exhausted by long perpetuation of vitiated constitutions, or long continuance of degrading and vicious habits.

Idiots and imbeciles are increasing in numbers at a rate greater than that of the general population. We also find that the present generation is more susceptible to stimulants and narcotic drugs, and that there is a great increase of the nervous diseases known as inebriety, hay fever, neuralgia, nervous dyspepsia, and nervous exhaustion or neurasthenia.

Idiocy has always been, until within a few years, classified with insanity, but the two have no similarity, except in the fact that each represents an abnormal condition of the brain.

Esquirol, who classified idiocy with insanity, wrote that "the condition of a man in a state of dementia may change; that of an idiot is ever the same."

Dr. Seguin says that not one in a thousand has been entirely refractory to treatment; not one in a hundred who has not been made happy and healthy; more than thirty per cent. have been taught to conform to social and moral law, and rendered capable of order, of good feeling, and of working like the third of a man; more than forty per cent. have been capable of the ordinary transactions of life under friendly control, of understanding moral and social abstractions, of working like two-thirds of a man; and twenty-five per cent. come nearer and nearer to the standard of manhood, till some of them will defy the scrutiny of good judges when compared with ordinary young women and men.

Goltz found, in his recent investigations, that when he removed a great portion of the hemisphere on one side, the animal did not become idiotic or demented; but if he removed portions from either hemisphere, the animal showed a diminution of mental power, with loss of tactile sensibility and awkwardness in its movements. Dr. Ireland, therefore, supposes that in idiots who improve under instruction, where the whole cerebrum is diseased, it in great part recovers its tone by being brought into healthy exercise, and that where a part still remains sound, it is thrown into more vigorous exercise than the rest, attracts a greater supply of blood, and gains a more vigorous nutrition than the surrounding parts.

Where the deficiency is congenital, the prognosis is often better than where it is traceable to diseases occurring in childhood; great improvement in the intelligence often follows improvement in the general health, as in the successful treatment of strumous and anæmic conditions.

Without training and education, idiots are an unproductive class; with it, their industrial capacity is greatly increased. The most zealous efforts of earnest teachers in the ordinary schools are ineffectual to meet the requirements of idiots, and it is only by teachers specially skilled and qualified for the duty that these unfortunates can be benefitted, and under their care, in many cases, astonishing moral, intellectual and physical improvement can be accomplished.

The greatest difficulty of the teacher is to properly classify his pupils and to form an estimate of their mental power and guess at what stage to begin, what faculties can be most readily called into cultivation, and what most require training and exercise. The difficulty of individualization is the great objection to asylums. We know that the blind and deaf are rendered less educable by associating with others having the like infirmity. So with feeble-minded children, where only a few are associated together they can receive better care; and kind treatment, good diet and attention will improve the most hopeless cases. Their teaching requires judgment, study and experience, and must be modified to suit each particular case.—*Chas. H. S. Davis, M.D., in Va. Med. Monthly.*

---

THERAPEUTIC EFFECTS OF HYOSCYMINE.—The following formula for its preparation was brought into use, and has been found to meet every requirement as to strength, but lacks stability: Hyoscyamine (Merck's crystaline), four grains; glycerine, distilled water, of each half an ounce; carbolic acid, two minims; dissolve without heat. Dose, four to eight minims, given hypodermically.

It is very important to make the solution without heat, as heat renders the alkaloid nearly inert. Hyoscyamine is a most unstable alkaloid, and soon decomposes; so that the strength of any solution yet devised cannot be depended on for more than one month after its preparation.

1. The observations show the uncertainty of the action of hyoscyamine when given by the mouth, and the danger of large doses.

2. They also show the marked superiority of the hypodermic method, and the confidence with which, in some cases, its effects could be calculated on, and the dose increased or diminished in accordance with the violence of the patient.

3. In hyoscyamine, we have a drug which is often capable of controlling the violence of a furious maniac, and, it may be, checking the torrent of rushing ideas on which he is borne along, soothing without putting him to sleep, and, in these respects, differing from morphia or chloral. In noisy and destructive general paralytics, the quiet air of comfort and repose following a moderate dose was such a contrast with the previous condition as to strongly impress every one with the feeling that, by the introduction of hyoscyamine, another valuable aid had been secured in the care and treatment of such cases.

4. No curative action can be claimed for the drug. Even in acute mania it did nothing more than moderate or check, for a time, the violence of action, and, perhaps, render less vivid and overwhelming the terrifying whirlwind of delusion of the frantic patient.

Previously to procuring a supply of hyoscyamine, the ordinary tincture of hyoscyamus was given in large doses, sometimes as much as one ounce at a time, without much effect, except that of dilating the pupils; but it had no controlling power in cases which afterwards yielded to the more powerful alkaloid.—*Thomas Browne, M D., in British Med. Jaurnal.*

---

Excision of Chancre as a Means of Aborting Syphilis.—In a paper read before the New York Academy of Medicine by Dr. P. L. Morrow the following were some of the conclusions reached: First, that the facts of clinical experience, as well as deductions from analogy and experience, were opposed to the theory of the local nature of chancre upon which the practice of excision was based. Second, that the practice of excision of chancre as a means of aborting syphilis was condemned by its clinical results when these results are weighed in the balance of a discriminating judgment, due regard being had to the possibility of error. Again, there was no evidence that the excision of chancre modified the constitutional symptoms of syphilis, if they appeared, by making them milder. Nor could the practice, as a means of treating a local sore, be considered in harmony with the principles of sound surgery, since, if left alone, the induration would undergo spontaneous absorption, and thus avoid the occurrence of a cicatrix.

In the subsequent discussion, Dr. E. L. Keyes said the author of the paper had treated of the subject under discussion so exhaustively, and had so nearly expressed his own views that it was unnecessary for him to add anything thereto. He did not believe that syphilis at the present time was so severe as it was in the fifteenth century. He read a part of a letter received from a physician who had been a resident of the Sandwich Islands for a number of years, in which it was stated that the population of those islands when the first census was taken, forty years ago, was 110,000, while to day it was only about 40,000. This rapid falling off in the population was supposed to be due to the effect of syphilis introduced among a virgin people at the time of the discovery of the islands by Captain Cook. At that time the disease was unknown among this people. The custom of the natives was such as to favor a rapid extension of syphilis. It would be considered an insult among them if a man staying all night at a friend's house were not invited to sleep with the wife, nor could a woman refuse any man the liberty of her person without offering him an insult. In those people, however, among whom syphilis had existed for generations, the affection, according to Dr. Keyes's observations, proved to be almost uniformly of a mild type.

He did not believe in the efficacy claimed for excision.—*Philadelphia Med. Times.*

---

INSTRUCTIONS TO GERMAN MIDWIVES.—1. Medical science has, for a number of years past, arrived at the fixed conviction that the great majority of cases of childbed fever depend for their origin upon the inoculation of recent wounds (produced more or less in every case of labor) by putrid (so-called septic) poisons.

2. These poisons readily cling—but in an imperceptible manner—to every kind of utensil that may come into contact with the woman in labor unless they are kept scrupulously clean.

3. But it is the hands and instruments of the obstetrician that have become known to be especially dangerous bearers and disseminators of the poisons, as it is impossible to avoid bringing these into contact with dangerous putrefying material.

4. Simple, and even repeated, washings with soap and water, as observation has shown, will not remove the poisons that may be clinging to the hands and instruments. The slightest trace of such a poison will, however, suffice to set up the gravest—yea, even fatal—childbed fever.

5. Wherefore it is the most sacred duty of every midwife, as of every physician, *in other ways* so thoroughly to cleanse the hands and instruments, and whatever other appliances may be brought into contact with the patient, that she may not be inoculated by any poisonous matter.

6. We are able, as we learn by observation, to destroy the poison, or, at least, render it powerless, by boiling water, and especially by the stronger solutions of carbolic acid (two to five per cent.).

7. The simple rising of the hands in carbolized solutions that are too weak can afford no protection against childbed fever. We therefore give, in the following, instructions as to the rational employment of the antiseptic material before, during, and after labor.—*Med. Press and Circular.*

---

A WINTER COLD.—1. A cold is usually ushered in by chilly sensations with fever, and if possible we should abort it at this stage by quinine, hot drinks and a sweat.

2. Failing this, the first stage of the inflammatory process which results from the cold is one of congestion and dryness. This cannot be arrested as a rule, and we therefore give expectorants internally, and make mildly stimulant local applications by inhalation and otherwise in order to promote the progressive evolution of symptoms.

3. The membrane having been relieved by the secreting stage setting in, we now control the process by using mild anodynes internally and making applications of local astringents.

4. If a cold relapses, or as it is said, "tightens up" again, we revert to our expectorants and local stimulants. —*F. H. Bosworth, M.D., in Independent Practitioner.*

---

Direct Mensuration of the Chest.—1. The relative expansion is interfered with early in disease.

2. In phthisis the measurement through the diseased lung after expiration is often greater than a corresponding measurement through the unaffected lung.

3. The measurement at corresponding points after forced inspiration is always less on the diseased side.

4. Age, sex and habits of life may modify the amount of chest expansion, but only disease can make any marked difference in the movements of the two sides.

5. Disease may be suspected where the difference in expansion at the apices is very small.

If these statements are true, comparative chest measurement is worthy of attention. It is important to know (1) the amount of movement; (2) the relative expansion; and (3) to have an accurate record by which to determine the future progress of the case.—*William Porter, M.D., in Medical Herald.*

---

Venereal and Common Warts.—Unna recommends for the treatment of condylomata acuminata and ordinary warts the continuous application of unguent. hydrarg. containing five per cent. of arsenic. In the case of a young girl upon whose hands were a hundred or more warts, the unbroken application for three weeks of a plaster containing in each 0.2 square metre 10.00 grammes of arsenic and .5 grammes of mercury, caused entire disappearance of the disease without any irritation of the normal skin. Cure was effected not by reason of necrosis and destruction of the warts, as after the use of caustics, but by resorption, as in cases of spontaneous cure.—*Translation by G. H. Tilden, M.D., in Boston Med. and Surg. Journal.*

---

MORAL INSANITY AND IMBECILITY.—Magnan (*Journal de medecine et de chirurgie pratiques*, April, 1882) reports a case of moral insanity of a distinctly marked type, which is sufficiently distinctive and clearly outlined to dispose of the cant that "moral insanity is unknown to medical science," which, at the Guiteau trial, emanated from a physician who was made an alienist expert during the journey from New York to Washington, by being diligently coached by a lawyer. The question of moral imbecility has some light thrown upon it by certain instructions given by Voisin (*Bulletin generale de therapetique medicale et chirurgicale*, March 30, 1882), who claims that affection, benevolence, and shame are very frequently absent among idiots and backward children, and only to be developed by careful tuition.—*Journal of Nervous and Mental Diseases.*

---

IODINE IN ERYSIPELAS.—Hutchinson records a case (*Brit. Med. Jour.*) of a large, robust man, thought to be *in extremis* from a violent attack of idiopathic erysipelas of head and face. Iron and usual internal treatment had been used but no local applications. Iodine was now painted on the scalp, which had a magical effect, amendment being immediate, and the patient being out of danger two days later. He had never seen so desperate a case recover so quickly. According to *Neale's Digest* the value of iodine has been known in this affection for thirty years.—*Maryland Med. Journal.*

